# Correlation between blood biomarkers and neuropsychiatric symptoms: Siriraj cohort, Thailand

**DOI:** 10.1002/alz.088520

**Published:** 2025-01-09

**Authors:** Vorapun Senanarong, Chatchawan Rattanabannakit, Witsarut Nanthasi, Natthamon Wongkom, Pathitta Dujada, Paphawadee Phoyoo, Lertchai Wachirutmangur

**Affiliations:** ^1^ Faculty of Medicine Siriraj Hospital, Mahidol University, Bangkok Thailand; ^2^ Division of Neurology, Department of Medicine, Faculty of Medicine Siriraj Hospital, Mahidol University, Bangkok Thailand

## Abstract

**Background:**

We explored the relationship of neuropsychiatric symptoms (assesses by NPI) and Alzheimer disease pathophysiology from blood AB42/40, GFAB, NFL, and pTau181. We also investigated if age and cognition were related to these neuropsychiatric symptoms.

**Method:**

222 subjects included 96 dementia, 66 MCI, and 60 normal controls (NC). Spearman correlation was used to calculation the correlation coefficient. Mann‐Whitney U test is used to compare biomarkers of those with and without subitem symptoms of NPI.

**Result:**

Mean ages were 71.26±9.82 years old in dementia group, 68.83±8.01 years old in MCI group, and 59.28±9.22 years old in NC group. Sum of 12 NPI subscale scores showed significant low correlation (r=0.20‐0.29) were correlated with blood AB42/AB40, Ptau 181, NFL, and GFAB. Sum of 12 NPI subscale scores showed significant moderate correlation with age and global cognition (assessed by Thai mental state examination, TMSE). Blood pTau181 displayed low correlation with hallucination, depression, anxiety, apathy, and irritability subscale scores. There was a significant low correlation between blood AB42/AB40 and delusion and irritability subscale scores. Blood NFL showed low correlation with euphoria, a, apathy, disinhibition, irritability, and aberrant motor activity. Inflammation process appeared to be significantly related with psychosis, apathy, disinhibition, and irritability. After controlled by age and TMSE; only apathy and AMA NPI subscale scores were correlated to NFL and GFAB accordingly.

**Conclusion:**

We discovered that psychosis and mood factors of NP symptoms were related to blood pTau. While most frontal factors of NP symptoms showed significant relationship with axonal destruction and microglia activation/inflammation.

This study was supported by grants from Health Systems Research Institute (HSRI), Thailand.

